# A Rare Presentation of Chronic Appendicitis in the Right Upper Quadrant: A Case Report

**DOI:** 10.7759/cureus.40772

**Published:** 2023-06-21

**Authors:** Ko-Han Chao, Chien-Yi Lin, Chih-Tang Wang

**Affiliations:** 1 Department of Internal Medicine, Lo-Sheng Sanatorium and Hospital, Ministry of Health and Welfare, New Taipei, TWN; 2 Department of Medical Imaging, Lo-Sheng Sanatorium and Hospital, Ministry of Health and Welfare, New Taipei, TWN; 3 Department of Surgery, Lo-Sheng Sanatorium and Hospital, Ministry of Health and Welfare, New Taipei, TWN

**Keywords:** atypical appendicitis, appendicolith, chronic abdominal pain, upper abdominal pain, chronic appendicitis

## Abstract

Chronic appendicitis is a rare cause of chronic abdominal pain that can be difficult to diagnose. We present a patient with chronic right upper quadrant pain that was finally diagnosed as chronic appendicitis. This 71-year-old male had no systemic diseases and presented to our outpatient clinic with right upper quadrant pain for one month. The pain tended to worsen in the early morning but could be relieved by bowel movements, sitting up, or walking. The findings of a physical examination, laboratory data, and abdominal ultrasound were not significant. Upper endoscopy revealed a shallow gastric ulcer at the antrum. However, the abdominal pain was not relieved by esomeprazole. A computed tomography (CT) scan revealed a dilated appendix with some appendicoliths in the retrocecal region. Due to chronic appendicitis, the patient underwent laparoscopic appendectomy, and the histopathological examination of the removed appendix confirmed the diagnosis. The abdominal pain completely resolved after the surgery. Chronic appendicitis should be kept in mind in patients with chronic abdominal pain without a definite diagnosis. This case illustrates that in addition to right lower quadrant pain, chronic appendicitis can also present with right upper quadrant pain or vague abdominal pain. A CT scan is invaluable in the diagnosis of abdominal pain when medical treatment fails to yield improvement.

## Introduction

Chronic appendicitis, which is characterized by the presence of fibrosis and chronic inflammation in pathological findings, is a rare cause of chronic abdominal pain that can be difficult to diagnose [1]. The incidence of chronic appendicitis has been reported to be 1% of all cases of appendicitis [2]. Herein, we present the case of a 71-year-old male with chronic retrocecal appendicitis with an atypical presentation and highlight the importance of computed tomography (CT) in diagnosing the condition.

## Case presentation

A 71-year-old male presented to our outpatient clinic with right upper quadrant pain for one month. He had no previous medical history. The patient described the pain as intermittent, with a dull and non-radiating nature, rated at “6/10” in severity. The pain lasted 1-2 hours and typically worsened in the early morning but could be alleviated by bowel movements, sitting up, or walking. A physical examination revealed no tenderness or rebound pain. Laboratory results showed mildly elevated alanine transaminase at 56 U/L (normal values: <35 U/L), while the leukocyte count, hemoglobin, thrombocytes, renal function, liver enzymes, and C-reactive protein were all normal. An abdominal ultrasound revealed a normal-appearing liver, gallbladder, and pancreas.

**Figure 1:**
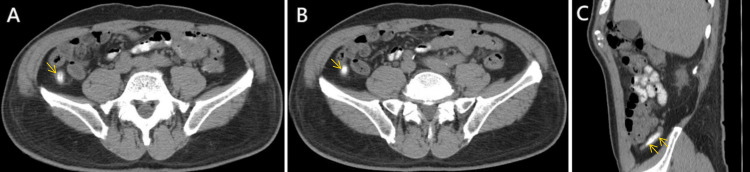
Computed tomography scan A and B: Transverse view showing thickening of the appendiceal wall and a dilated appendix with appendicoliths (yellow arrows). C: Sagittal view showing the retrocecal location of the appendix (yellow arrows).

Upper endoscopy revealed a shallow gastric ulcer at the antrum. Esomeprazole was prescribed, which provided mild pain relief. However, the patient experienced recurrent abdominal pain one week later and was subsequently admitted to our hospital. Laboratory results showed a normal white blood cell count and C-reactive protein level. A CT scan revealed a thickening appendiceal wall and dilated appendix with some appendicoliths in the retrocecal region (Figure 1). As a result, he underwent a laparoscopic appendectomy. A histopathological examination of the removed appendix confirmed chronic inflammation and fibrosis, consistent with chronic appendicitis. Following the surgery, the abdominal pain and other symptoms completely resolved.

## Discussion

In most circumstances, the appendix is located on the posterolateral aspect of the cecum, 2 cm below the ileocecal valve. However, there are various atypical positions of the appendix, including retrocecal (65.3%), pelvic (31%), subcecal (2.3%), pre-ileal (1%), and post-ileal (0.4%) [3]. While right lower quadrant pain is the most common presentation of appendicitis, it is possible for appendicitis to present with right upper quadrant pain or dull abdominal pain in rare cases due to variations in anatomical structure or the location of the appendix, such as a retrocecal appendix [3-7]. Retrocecal appendicitis presenting with right upper abdominal pain may be clinically indistinguishable from other pathologies in the gallbladder, liver, biliary tree, right kidney, and right urinary tract [8]. This emphasizes the importance of performing CT early in patients with refractory abdominal pain. To the best of our knowledge, this is the first case report of chronic appendicitis with a retrocecal location presenting with right upper quadrant pain.

There are currently no standard diagnostic criteria for chronic appendicitis, and an early diagnosis is difficult due to its rarity and occasionally atypical presentation of pain [9]. The diagnosis is often made after appendectomy with compatible histological findings of chronic inflammatory changes [1]. A previous study reported that a preoperative period of pain of seven days was the optimal cutoff value to indicate histologically non-acute appendicitis with a high specificity and high positive predictive value [10]. Another study reported that persistent right lower quadrant pain for at least six months and typical imaging findings could be used to make a preliminary diagnosis of patients with suspected chronic appendicitis [11]. However, we favor a cutoff value of seven days due to the potential complications of chronic appendicitis, including perforation and peritonitis [12].

CT findings of chronic appendicitis include the presence of appendicoliths and appendiceal thickening but a lack of mesenteric infiltration or abscess [13]. The CT presentation in our case showed both appendiceal thickening and appendicoliths. However, appendicoliths are commonly seen in both asymptomatic and symptomatic patients [14]. The presentation of appendicoliths alone is not a reliable feature to diagnose appendicitis.

## Conclusions

In conclusion, chronic appendicitis is a rare disease that may present with right upper quadrant pain and can be difficult to diagnose. This case highlights the importance of arranging CT as early as possible in patients with abdominal pain of unknown etiology, even without classic signs of acute appendicitis. Classic CT findings of chronic appendicitis include the presence of appendicoliths and appendiceal thickening but a lack of mesenteric infiltration or abscess. Early diagnosis and treatment of chronic appendicitis may prevent the development of potentially life-threatening complications.
